# Supplementary material for: Multivalent ion-mediated nucleic acid helix-helix interactions: RNA versus DNA
Source: Nucleic Acids Res. 2015 May 27;43(12):6156–65. doi: 10.1093/nar/gkv570 (PMC4499160; doi:10.1093/nar/gkv570)
Supplement: SUPPLEMENTARY DATA [file supp_43_12_6156__index.html]

Multivalent ion-mediated nucleic acid helix-helix interactions: RNA versus DNA — Multivalent ion-mediated nucleic acid helix-helix interactions: RNA versus DNA — SUPPLEMENTARY DATA 

# Multivalent ion-mediated nucleic acid helix-helix interactions: RNA versus DNA

## SUPPLEMENTARY DATA

- SUPPLEMENTARY DATA
