# Supplementary material for: Multivalent ion-mediated nucleic acid helix-helix interactions: RNA versus DNA
Source: Nucleic Acids Res. 2015 May 27;43(12):6156–65. doi: 10.1093/nar/gkv570 (PMC4499160; doi:10.1093/nar/gkv570)
Supplement: SUPPLEMENTARY DATA [file supp_gkv570_nar-00875-f-2015-File008.pdf]

Supplementary Material for

Multivalent ion-mediated nucleic acid helix-helix interactions: RNA versus DNA

Yuan-Yan Wu[1], Zhong-Liang Zhang[1], Jin-Si Zhang[1], Xiao-Long Zhu[2] and Zhi-Jie Tan[1*]

[1]*Department of Physics and Key Laboratory of Artificial Micro & Nano-structures of Ministry of Education, School of Physics and Technology, Wuhan University, Wuhan 430072, China*

[2]*Department of Physics, School of Physics & Information Engineering, Jianghan University, Wuhan 430056, China*

[*]To whom correspondence should be addressed: zjtan@whu.edu.cn

To get desirable bulk Co-Hex and Na^+ concentrations

Due to the competition between monovalent and multivalent ions in binding to nucleic acids (e.g., Ref. (29)), it is not straightforward to obtain desirable bulk monovalent/multivalent ion concentrations in a MD simulation for a nucleic acid in a mixed monovalent/multivalent ion solution.

In the present work, to get the desirable bulk ion concentrations, before the all-atom MD simulations, the simplified Monte Carlo (MC) simulations (50,66) are employed to estimate numbers of Co-Hex and Na^+ ions in the simulational cell. Practically, all-atom structure of nucleic acids and ions are placed in the MC simulational cell, and water is treated as continuous medium with dielectric constant of 78 (50,66). The MC algorithm with Coulomb and Lenard-Jones potentials (50,66) is performed to get the bulk ion concentrations in equilibrium. We change the relative numbers of Co-Hex and Na^+ ions and repeat the MC processes, and then we can estimate the numbers of Co-Hex and Na^+ ions in the simulational cell at the desirable bulk ion concentrations. Afterwards, the numbers of Co-Hex and Na^+ ions from the simplified MC simulations are used in the all-atom simulations, and the realistic bulk Na^+ and Co-Hex concentrations from the all-atom MD simulations are very close to the desirable values; see Fig. S1 in Supplementary Material for the cases of 100mM Na^+/5mM Co-Hex solutions.

Osmotic pressure for DNA aggregates and comparison with experimental data

For DNA array (DNA aggregates), the osmotic pressures have been measured experimentally by the osmotic stress technique. In this section, based on the pair-wise DNA helix-helix interactions, we have calculated the osmotic pressures and compared the results with the experimental data. For a hexagonal DNA array (see Fig. 11a in Ref. (46)), the mean free energy Δg(x) per DNA can be approximately calculated through the summation over the pair-wise helix-helix interactions between nearest neighbor pairs (46,78)

$$\Delta g(x) = \sum_{i=1}^{6} \Delta G_i(x)/2, \tag{S1}$$

where $\Delta g(x) = \sum_{i=1}^{6} \Delta G(x)$ is the total free energy between an helix and its six neighbors, and the factor 1/2 is used to remove double-counting. $\Delta G_i(x)$ is the free energy for the two-helix system of a helix and its i-th neighbor. Here, we have neglected the nonadditive effect in multiple helix packing and kept only interactions between the nearest neighboring helices, i.e., when calculating the interaction between two helices, we ignore the existence of other helices.

The osmotic pressure Π(x) as a function of the helix-helix distance x can be calculated from (46,78)

$$\Pi(x) = -\frac{\Delta g(x)}{\partial V}, \tag{S2}$$

where *V*=*L*×*A* is the volume of the hexagonal region around each helix. *L* is the length of each helix, and $\sqrt{3}x^2/2$ A=3 is the average cross section area per molecule in the DNA array (46). Practically, we first fit the calculated PMF to a polynomial function and based on the fitted PMF, we could easily calculate the osmotic pressures according to Eqs. S1 and S2.

As shown in Fig. S8, for DNAs in 5mM Co-Hex/100mM Na^+, the calculated osmotic pressures are very close to the experimental data (15,16), and the slight deviation may come from the multi-helix effect (46) which was ignored in our calculations for hexagonal DNA aggregate. For DNAs in 0.5mM Co-Hex/100mM Na^+, there is no directly available experimental data. As shown in Fig. S8, the addition of 0.5mM Co-Hex in 100mM Na^+ can cause a different osmotic pressure curve from that for 100mM Na^+ (Ref. (1) in supplementary material).

Table S1 A-RNA, B-DNA and A-DNA sequences used in the present study.[a]

Nucleic Acids	Sequences
A-RNA	5’-CGACUCUACUACGCGC-3’<br>GCUGAGAUGAUGCGCG
B-DNA	5’-CGACTCTACTACGCGC-3’<br>GCTGAGATGATGCGCG
A-DNA	5’-CGACTCTACTACGCGC-3’<br>GCTGAGATGATGCGCG

[a]The sequences of the short RNA and DNAs are selected according to the recent small angle scattering experiments and UV measurements (29).

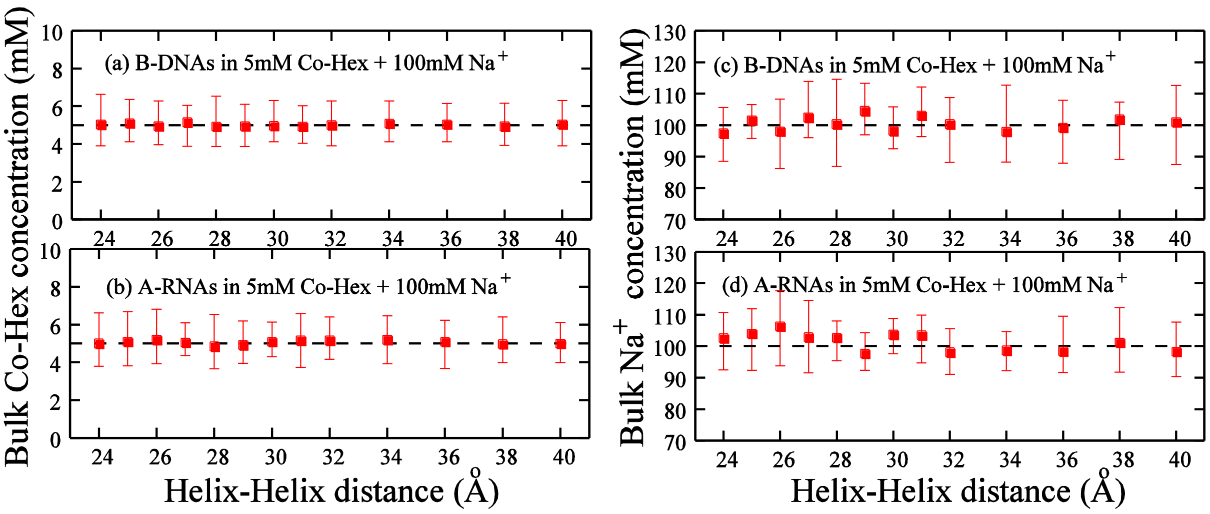

Figure S1 (a,c) The realistic bulk Co-Hex (a) and Na^+ (c) concentrations for two DNAs in 100mM Na^+/5mM Co-Hex solutions from the all-atom MD simulations as a function of DNA-DNA distance; (b,d) The realistic bulk Co-Hex (b) and Na^+ (d) concentrations for two RNAs in 100mM Na^+/5mM Co-Hex solutions from the all-atom MD simulations as a function of RNA-RNA distance.

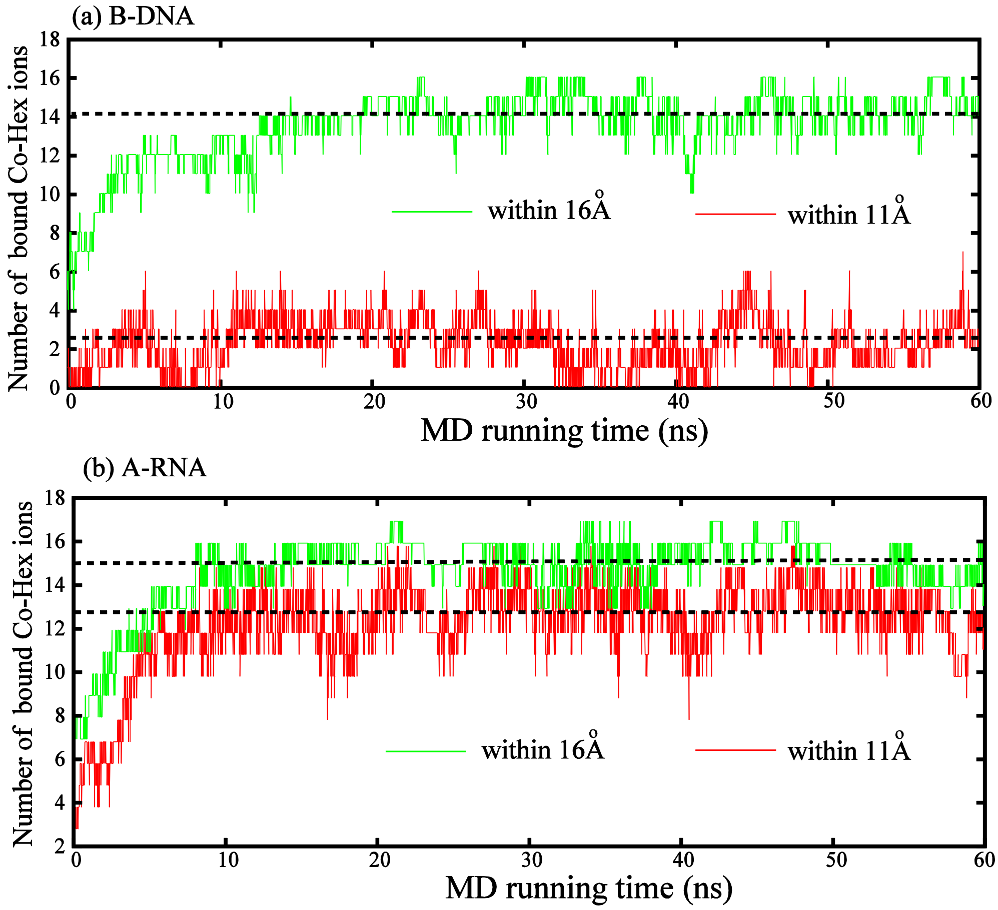

Figure S2 Number of bound Co-Hex ions within the distances of 11Å (red) and 16Å (green) from two duplex helical axes as function of MD running time: (a) B-DNAs and (b) A-RNAs at 5mM Co-Hex and 100mM Na^+ ion solution. Dashed lines denote the averaged values in equilibrium.

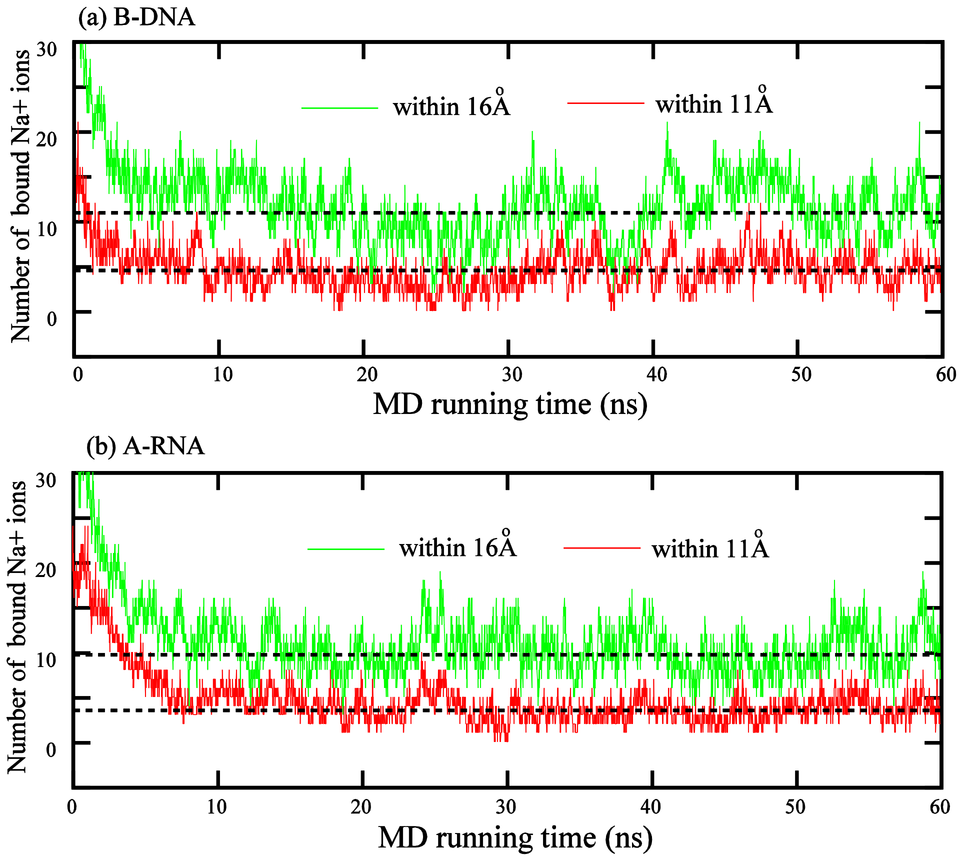

Figure S3 Number of bound Na^+ ions within the distances of 11Å (red) and 16Å (green) from two duplex helical axes as function of MD running time: (a) B-DNA and (b) A-RNA at 5mM Co-Hex and 100mM Na^+ ion solution. Dashed lines denote the averaged values in equilibrium.

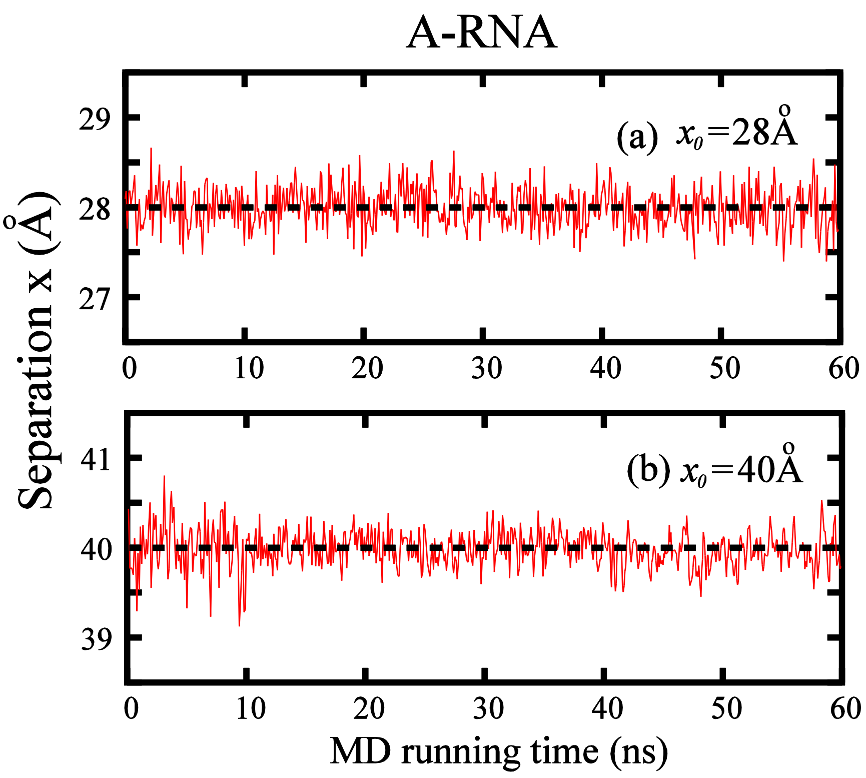

Figure S4 An illustration of the MD convergence of separation x between the centers of mass of two 16-bp A-RNA helices in 5mM Co-Hex ion solution with 100mM NaCl. The averaged values of separation x are made over every Δt by $\int_{t-\Delta t/2}^{t+\Delta t/2} x(t')dt'/\Delta t$ and Δt =20ps. The black lines denote the averaged values in equilibrium. Two typical original separations x_0 between two A-RNAs are shown in the panels.

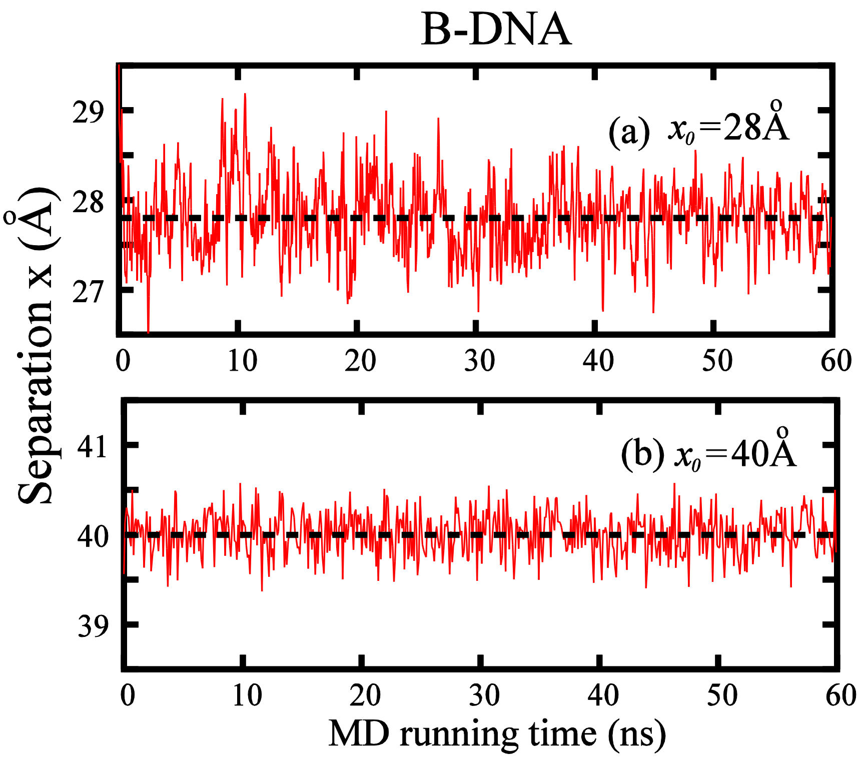

Figure S5 An illustration of the MD convergence of separation x between the centers of mass of two 16-bp B-DNA helices in 5mM Co-Hex ion solution with 100mM NaCl. The averaged values of separation x are made over every Δt by $\int_{t-\Delta t/2}^{t+\Delta t/2} x(t')dt'/\Delta t$ and Δt =20ps. The black lines denote the averaged values in equilibrium. Two typical original separations x_0 between two A-DNAs are shown in the panels.

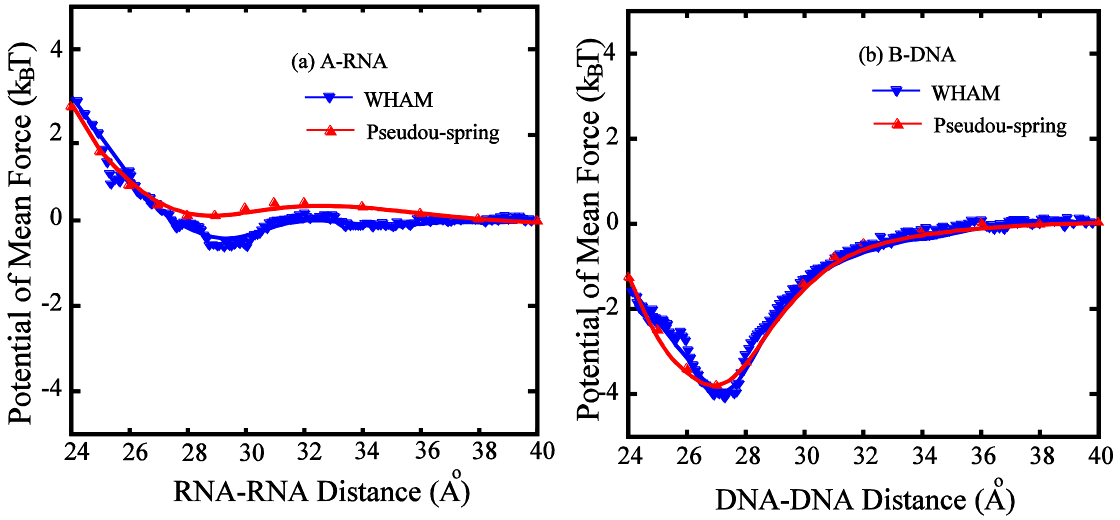

Figure S6 The potential of mean force as a function of the separation *x* between the centers of mass of two 16-bp nucleic acid helices in 5mM Co-Hex ion solution with 100mM NaCl: (a) A-RNAs and (b) B-DNAs. Blue: calculated from the umbrella sampling with the weighted histogram analysis method (WHAM); Red: calculated from the pseudo-spring method employed in the present work.

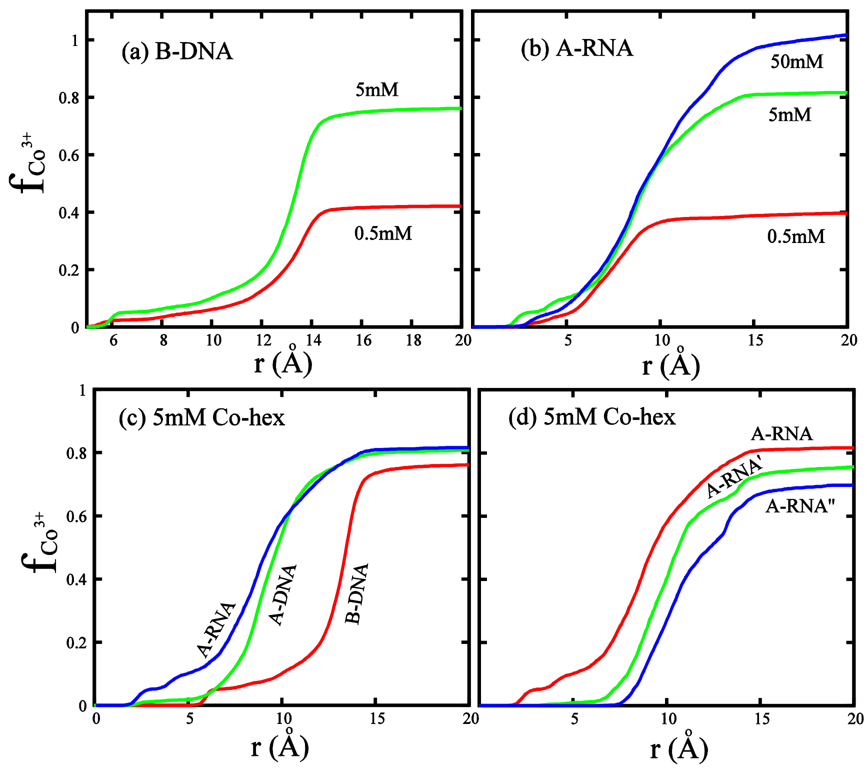

Figure S7 The Co-Hex charge distribution per unit charge on nucleic acid helices as a function of distance *r* from the axes of nucleic acid helices. (a) B-DNAs at 0.5mM and 5mM [Co-Hex]'s; (b) A-RNAs at 0.5mM, 5mM and 50 mM [Co-Hex]'s; (c) The comparisons between A-RNAs, B-DNA sand A-DNAs; (d) The modified A-RNAs. The A-RNA' denotes the A-RNA with the bottom of the central 1/3 major groove fixed with a layer of water, whereas the A-RNA" denotes the A-RNA with the bottom of the entire major groove fixed with a layer of water. Please note that the buffers always contain 100mM NaCl.

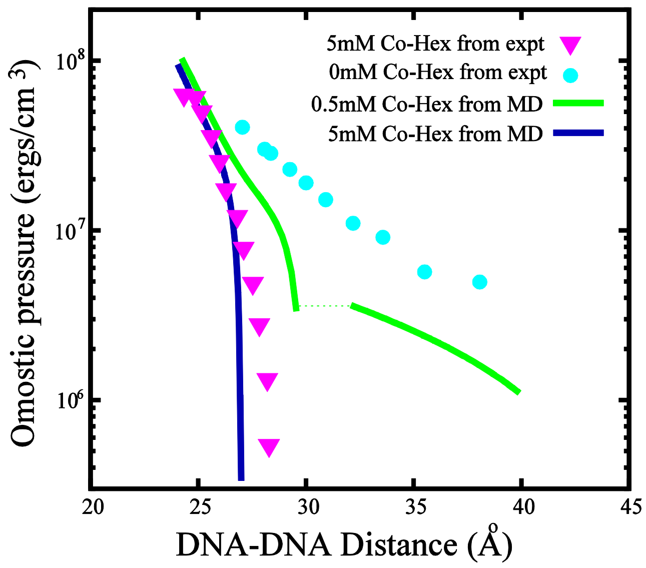

Figure S8 Comparison of osmotic pressure curves for hexagonal DNA aggregate in various salt solutions from MD simulations (lines) and experiments (symbols). Lines: DNAs at 0.5mM (green) and 5mM (blue) [Co-Hex]'s, and the buffers always contain 100mM NaCl. Symbols: ▼, experimental data for DNAs at 5mM Co-Hex and 100mM NaCl with 10mM TrisCl (17); ●, experimental data for DNAs at 100mM NaCl with 10mM TrisCl (Ref. 1 in Supplementary Material). The apparent deviation between blue curve and ● comes from that the experiments did not involve Co-Hex ions.

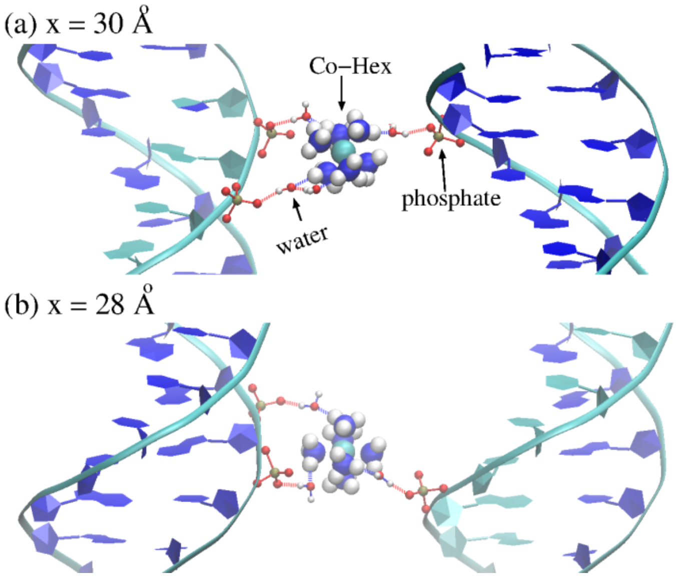

Figure S9 Two snapshots to illustrate the structure of water molecules which link the bridging Co-Hex and phosphates in two DNAs. The axis-axis distances between DNAs are 30Å (a) and 28 Å (b), respectively. The dash lines are H-bonds which are automatically displayed by the software VMD (Ref. 2 in Supplementary Material).

References

1. Rau, D. C, Lee, B., Parsegian, V. A. (1984) Measurement of the repulsive force between polyelectrolyte molecules in ionic solution: hydration forces between parallel DNA double helices. Proc. Natl. Acad. Sci. USA 81, 2621-5.
2. Humphrey, W., Dalke, A., Schulten K. (1996) VMD: visual molecular dynamics. J. Mol. Graphics, 14, 33-38.
